# The Impact of IDH1 Mutation and MGMT Promoter Methylation on Recurrence-Free Interval in Glioblastoma Patients Treated With Radiotherapy and Chemotherapeutic Agents

**DOI:** 10.3389/pore.2021.1609778

**Published:** 2021-04-29

**Authors:** Maher Kurdi, Nadeem Shafique Butt, Saleh Baeesa, Badrah Alghamdi, Yazid Maghrabi, Anas Bardeesi, Rothaina Saeedi, Taghreed Al-Sinani, Najla Alghanmi, Mohammed O. Bari, Alaa Samkari, Ahmed I. Lary

**Affiliations:** ^1^ Department of Pathology, Faculty of Medicine in Rabigh, King Abdulaziz University, Jeddah, Saudi Arabia; ^2^ Department of Family and Community Medicine, Faculty of Medicine in Rabigh, King Abdulaziz University, Jeddah, Saudi Arabia; ^3^ Division of Neurosurgery, Faculty of Medicine, King Abdulaziz University, Jeddah, Saudi Arabia; ^4^ Department of Physiology, Faculty of Medicine, King Abdulaziz University, Jeddah, Saudi Arabia; ^5^ Department of Neuroscience, King Faisal Specialist Hospital, Jeddah, Saudi Arabia; ^6^ Department of Surgery,Division of Neurosurgery, King Fahad General Hospital, Jeddah, Saudi Arabia; ^7^ Department of Pathology, King Abdulaziz University Hospital, Jeddah, Saudi Arabia; ^8^ Department of Pathology and Laboratory Medicine, King Saud Bin Abdulaziz University for Health Science, Jeddah, Saudi Arabia; ^9^ Section of Neurosurgery, Department of Surgery, King Abdulaziz Medical City, Jeddah, Saudi Arabia

**Keywords:** glioblastoma, chemotherapy, IDH1 mutation, MGMT promoter methylation, radiotherapy

## Abstract

The aim of this study is to investigate the relationship between isocitrate dehydrogenase-1 (*IDH1*) mutation and O^6^-methylguanine-DNA methyltransferase (*MGMT*) promoter methylation with recurrence-free interval in glioblastoma patients treated with chemoradiotherapies. Clinical data were collected from 82 patients with totally resected glioblastoma and treated with adjuvant therapies from 2014 to 2019. *IDH1* mutation was assessed by immunohistochemistry and *MGMT* promoter methylation was assessed by different sequencing methods. *IDH1* mutation was present in 32 cases and 50 cases were *IDH1* wildtype; 54 and 28 patients had unmethylated and methylated *MGMT* promoter, respectively, Of the 82 patients, 62 patients received chemoradiotherapy while 20 patients only received radiation. Approximately, 61% of patients had a tumor recurrence after 1 year, and 39% showed a recurrence before 1 year of treatment. There was no significant relationship between *IDH1* mutation and *MGMT* promoter methylation (*p*-value = 0.972). Patients with *IDH1* mutation and their age <50 years showed a significant difference in recurrence-free interval (*p*-value = 0.014). Difference in recurrence-free interval was also statistically observed in patients with unmethylated *MGMT* promoter and treated with chemoradiotherapies (*p*-value = 0.031), by which they showed a late tumor recurrence (*p*-value = 0.016). This revealed that *IDH1* mutation and *MGMT* methylation are independent prognostic factors in glioblastoma. Although *IDH1*-mutant glioblastomas showed late tumor recurrence in patients less than 50 years old, the type of treatment modalities may not show additional beneficial outcome. Patients with unmethylated *MGMT* and *IDH1* mutation, treated with different chemoradiotherapies, showed a late tumor recurrence.

## Introduction

Glioblastoma is the most aggressive primary malignant brain tumor in adults [1]. The current standard treatment for glioblastoma after surgical resection is combined radiotherapy and chemotherapy using temozolamide (TMZ) or TMZ combined with other chemotherapeutic agents. Despite these treatments, glioblastoma remains a fatal disease and the overall survival is approximately 14.6 months within five years [2, 3]. However, the current treatment strategies are considered palliative, with the aim being to improve patient survival and maintain a good quality of life. Several biomarkers are still used in clinical practice to improve the diagnostic and prognostic status of glioblastoma. One of these markers are IDH1^R132H^ mutation and *MGMT* gene promoter methylation. The *IDH1* gene, which encodes cytosolic NADP + -dependent isocitrate dehydrogenase, was shown to correlate with outcome of patients with glioblastoma [4–6]. The *MGMT*, which is a DNA repair protein that removes alkyl groups from several residues particularly the O6-position of guanine, is considered the most relevant for the action of an extensively used TMZ [7, 8]. Because IDH1^R132H^ mutation, is the most common variant mutation detected in most of cancers, particularly glioblastoma, we used specific monoclonal antibody (IMAB-1) targeted R131H residue.

The relationship of *MGMT* promoter methylation with *IDH1* mutation, treatment modalities and survival rates showed controversial results. Some studies revealed that glioblastomas with methylated *MGMT* promoter were more sensitive to chemotherapeutic agent (TMZ) resulting in a better survival rate [9–11], while other studies revealed that *MGMT* promoter methylation was not associated with better overall survival (OS) [12, 13]. Combs et al. also found a none-favourable OS in a group of 160 patients with glioblastoma treated with radiotherapy and TMZ. In contrast, Millward et al. demonstrated that the combination of methylated *MGMT* promoter and *IDH1* mutation was associated with considerably longer OS in a series of chemoradiotherapy-treated glioblastoma tumors [14]. Despite this controversy, both biomarkers are still considered prognostic factors in patients with glioblastoma.

In this study, we evaluated the impact of *MGMT* promoter methylation and *IDH1* mutation on the recurrence-free interval in patients with glioblastoma treated with different treatment modalities (radiotherapy and different types of chemotherapies). The chemotherapy protocol included temozolomide (TMZ) alone, or TMZ and other adjuvant therapies included (bevacizumab, irinotecan, lomustine, and etoposide). This study provided an additional justification to the previous controversial results in the literature. To our knowledge, this study is considered as a first research of such type performed in Saudi Arabian patients.

## Materials and Methods

### Patients and Stratification

**FIGURE 1:**
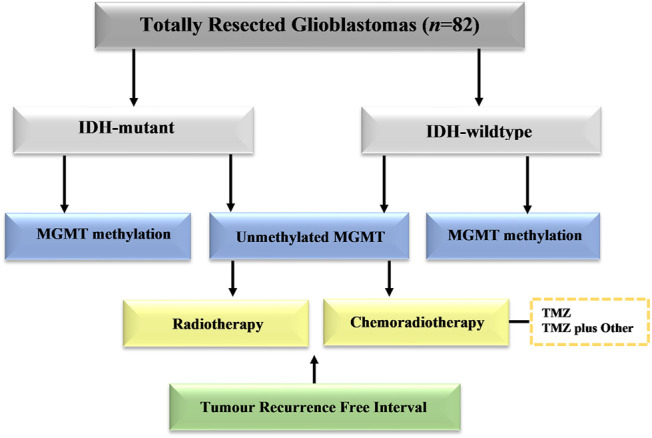
Schematic distribution of patient groups in the study group. Cases with totally resected glioblastoma were stratified into groups based on *IDH1* mutation, *MGMT* promoter methylation, and adjuvant therapies.

The study included 82 patients with completely resected glioblastoma from two medical institutions in Saudi Arabia in the period of 2014–2019. This study was approved by the National Biomedical Ethics Committee at King Abdulaziz University (HA-02-J-008) under a general ethical report. All patients underwent complete surgical resection of the tumor followed by a standard protocol of chemoradiotherapy. The histological diagnosis was made based on classification of the World Health Organization (WHO). Clinical data were retrieved from the hospital records and included patient age at diagnosis, gender, post-operative adjuvant therapies, *MGMT* methylation profile, and *IDH1* results. The patients were stratified into different groups based on the adjuvant therapies (Figure 1). Standard radiotherapy of a total dose of 60 Gy and TMZ (150–200 mg/m^2^ for 6–12 cycles) was given to all patients at the time of management. The chemotherapy protocol included temozolomide (TMZ) alone, or TMZ and other adjuvant therapies included (bevacizumab, irinotecan, lomustine, and etoposide). All patients enrolled in this study have passed away. Those who had late recurrence died because of other comorbidities. The only limitation should be clarified that the total number of cases analyzed in this study is still considered small. Despite this limitation, this is the first study in Saudi Arabia that explore the impact of MGMT methylation and IDH1 on tumour recurrence in patients receiving different type of treatment modalities.

### Assessment of IDH1 Mutation

*IDH1* mutation was evaluated by immunohistochemistry. Anti-*IDH1* ^R132H^ mouse monoclonal antibody (clone H09) was used to identify wild-type and mutant IDH1 in formalin-fixed paraffin-embedded (FFPE) sections using a BenchMark XT automated staining system from Ventana. Sections in which >10% of tumor cells positively stained were defined as *IDH1* mutant.

### Assessment of MGMT Promoter Methylation

*MGMT* promoter methylation was assessed in the hospitals at time of histopathological diagnosis by using two different techniques: methylation-specific polymerase chain reaction (MSP) or pyrosequencing method using Qiagen.

First method used: Qualitative methylation-specific PCR (MSP). The regions were selected from which DNA could be extracted. DNA extraction was performed using the QIAamp DNA FFPE tissue kit according to the manufacturer’s instructions. DNA quantity and quality were determined using a NanoDrop spectrophotometer at A260/A280 and A260/A230. The concentration of DNA samples was normalized to 50 ng and bisulfite-converted using the EpiTect Bisulfite Kit (Qiagen) according to the manufacturer’s instructions. The forward and reverse primers targeting methylated and unmethylated exon 1 of the human *MGMT* gene correspond to those described by Esteller et al. [15]. The PCR Kit used was HotStarTaq plus DNA polymerase (Qiagen). Thermal cycling on a Veriti thermal cycler included an initial step at 95°C for 2 min followed by 40 cycles of 30 s at 94°C, 30 s at 52°C, and 30 s at 72°C for 10 min. The PCR products were visualized on 8% non-denaturing polyacrylamide gels and stained with ethidium bromide. Samples having only methylated PCR products and samples having both methylated and non-methylated PCR products were both scored as methylation positive.

Second method used: Pyrosequencing technique. The MGMT pyro Kit from Qiagen has been used for quantitative measurement of methylation at four CpG sites in exon 1 of human MGMT gene (genomic sequence on chromosome 10 from 131,265,519 to 131,265,537 sequencing from 72 to 90 on MGMT mRNA. DNA extraction was performed using the QIAamp DNA FFPE tissue kit according to the manufacturer’s instructions. DNA quantity and quality were determined using a NanoDrop spectrophotometer. The concentration of DNA samples was normalized to 50 ng and bisulfite-converted using the EpiTect Bisulfite Kit (Qiagen) according to the manufacturer’s recommendation. The Thera screen MGMT PyroKit and PyroMark Q24 system were both used to assess the methylation status. DNA was amplified by PCR while single-stranded DNA was prepared, sequenced, and finally analyzed on PyroMark Q24. The methylated control DNA was included in the kit as a positive control for PCR and sequencing reaction. The procedure corresponds to what has been described by Pangopoulos et al [16].

### Statistical Methods

Data are described as frequencies and percentages. Pearson’s Chi-Square and Fisher’s Exact tests were used to explore the association of *IDH1* mutation, *MGMT* promoter methylation status, and chemotherapies with various study factors. Kaplan–Meier curves and log-rank test were used to compare the distribution of recurrence-free interval. Recurrence-free interval was defined as the period from the beginning of adjuvant therapy after surgical resection to the possible first date of recurrence. In survival analysis, the term “event” is usually used to measure the occurrence of an event of interest (such as death, recurrence, and recovery). In our study, the event of interest is “recurrence.” The “number of cases at risk” defines how many cases will be at risk in the end of a specified time point. Being “at risk” clarifies that the patient has not had a recurrence before a time and is not censored before or at time. Moreover, the number of events” measures that number of cases for which an event of interest is observed. All statistical analyses in this study were performed using IBM SPSS1 ver. 24 statistical software programs (SPSS Inc. Chicago, IL, United States).

## Results

A total of 82 glioblastoma patients with complete resection and who received treatment were included in this study. The patient and tumor characteristics of the study group are listed in Table 1. The mean patient age was 48 years old (<50 years old, *n* = 32; ≥50 years old, *n* = 50), and 58.5% of the study group was male (*n* = 48). *IDH1* mutation was found in 32 cases (39%) and the remaining 50 cases (61%) were *IDH1* wildtype. The *MGMT* promoter was methylated in 28 patients (34.1%) and unmethylated in 54 cases (65.9%). After complete tumor resection, 62 patients (75.6%) received chemoradiotherapy while 20 patients (24.4%) only received radiotherapy. Among those who received chemoradiotherapy, approximately 72.5% (*n* = 45) received TMZ alone while 27.5% (*n* = 17) received TMZ along with other chemotherapies included bevacizumab, irinotecan, lomustine, or etoposide. Approximately 61% (*n* = 50) had tumor recurrence after 1 year, while 39% (*n* = 32) showed recurrence before 1 year of the adjuvant therapies.

**TABLE 1:** Patient and tumor characteristics of the study group (*n* = 82).

Characteristic	*n* (%)
Age	
Mean	48.4
Age group	
<50 years	32 (39.0%)
≥50 years	50 (61.0%)
Gender	
Female	34 (41.5%)
Male	48 (58.5%)
*IDH1* mutation status	
IDH1 mutant	32 (39.0%)
IDH1 wildtype	50 (61.0%)
*MGMT* promoter methylation	
Methylation	28 (34.1%)
Unmethylated	54 (65.9%)
Adjuvant therapy	
Radiotherapy	20 (24.4%)
Radiotherapy and chemotherapy	62 (75.6%)
Chemotherapy	
Temozolomide	45 (72.5%)
Temozolomide plus others	17 (27.5%)
Recurrence	
Before 1 year	32 (39.0%)
After 1 year	50 (61.0%)

### IDH1 Mutation and MGMT Methylation in Glioblastoma Patients

Although wildtype *IDH1* was frequently found in glioblastoma patients with unmethylated *MGMT* promoter, there was no significant relationship between *IDH1* mutation and *MGMT* promoter methylation in the study group (*p*-value = 0.972) (Table 2).

**TABLE 2:** Relationship between *IDH1* mutation and *MGMT* promoter methylation in glioblastoma patients.

	*MGMT* methylation (*n* = 28)	Unmethylated *MGMT* (*n* = 54)	Total (*n* = 82)	*p*-value
*IDH* status				0.972^a^
*IDH1* mutant	11.0 (39.3%)	21.0 (38.9%)	32.0 (39.0%)	
*IDH1* wildtype	17.0 (60.7%)	33.0 (61.1%)	50.0 (61.0%)	

Data are shown as *n* (%).

^a^ Pearson's Chi-squared test.

### Relationship of Age and IDH1 Mutation or MGMT Methylation Status With Recurrence-Free Interval in Glioblastoma Patients

**FIGURE 2:**
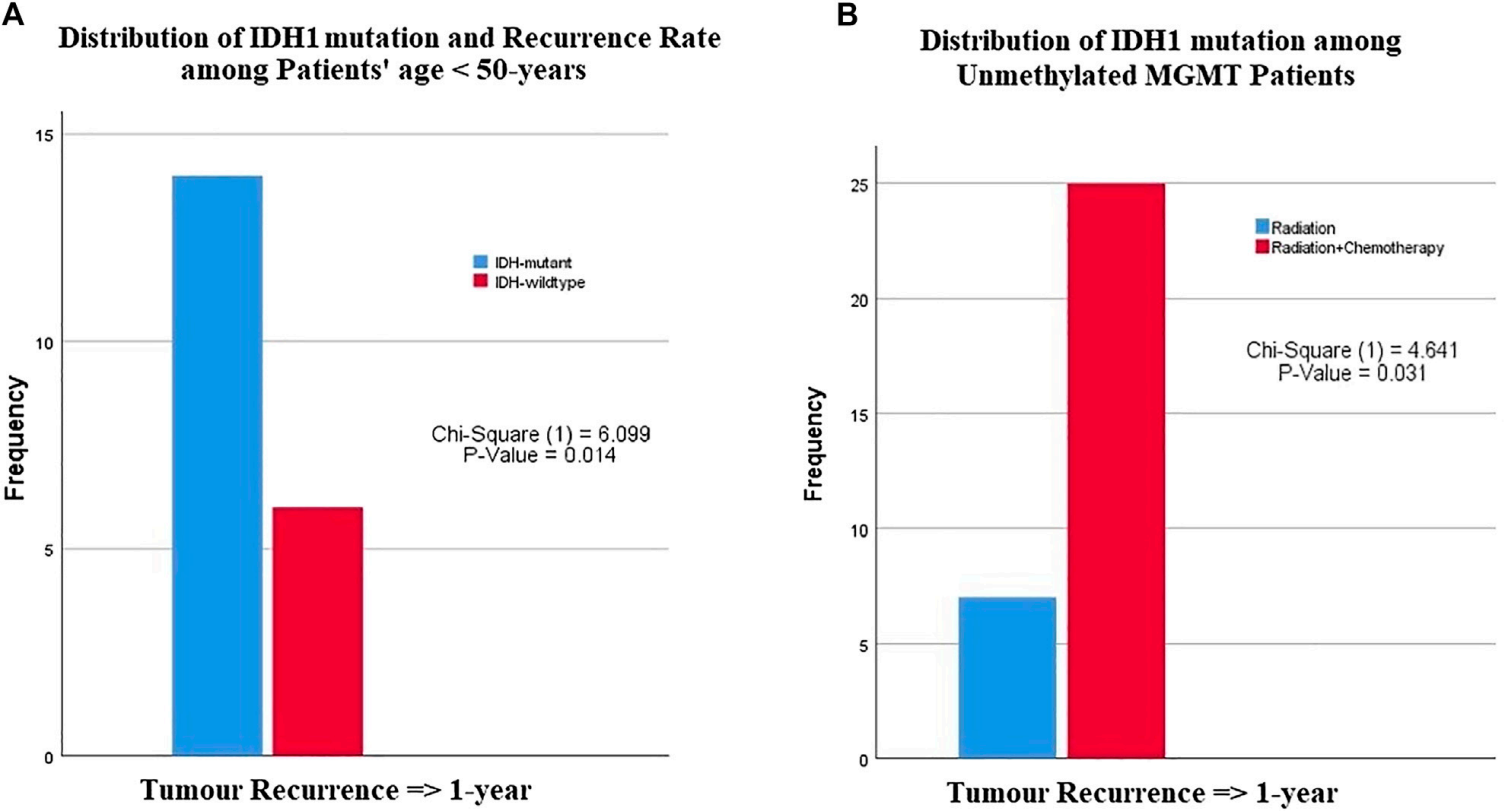
The relationship between *IDH1*-mutation status and *MGMT* promoter methylation with patients age, recurrence rate or type of chemotherapies. **(A)** Among patients younger than 50 years of age, patients with *IDH1* mutation had a late recurrence after one year compared with patients with wildtype *IDH1*, **(B)** patients with unmethylated *MGMT* promoter had a recurrence after 1 year of chemoradiotherapy compared with those who received only radiotherapy.

Among patients less than 50 years of age, a significant difference was observed in recurrence-free interval between glioblastomas with *IDH1* mutation and wildtype *IDH1* (*p*-value = 0.014) (Table 3). These results indicated that *IDH1* mutant cases showed a late recurrence rate compared with those with wildtype *IDH1* (Figure 2A). In contrast, patients over 50 years of age did not show any significant difference in recurrence-free interval, regardless of *IDH1* status. There was no significant difference in recurrence-free interval among glioblastoma patients based on age and *MGMT* promoter methylation status.

**TABLE 3:** The relationship between age, *IDH1* mutation, and *MGMT* promoter methylation in glioblastoma patients and one-year recurrence-free interval.

				Recurrence-free interval			
				<1 year		≥1 year		*p*-value^a^ ^,^ ^b^
Age	<50 years	*IDH1* status	*IDH1* mutant	3	(17.6%)	14	(82.4%)	0.014^a^
			*IDH1* wildtype	9	(60.0%)	6	(40.0%)	
	≥50 years		*IDH1* mutant	3	(20.0%)	12	(80.0%)	0.059^a^
			*IDH1* wildtype	17	(48.6%)	18	(51.4%)	
Age	<50 years	*MGMT* status	Methylation	5	(50.0%)	5	(50.0%)	0.438^b^
			Unmethylated	7	(31.8%)	15	(68.2%)	
	≥50 years		Methylation	5	(27.8%)	13	(72.2%)	0.188^a^
			Unmethylated	15	(46.9%)	17	(53.1%)	
*IDH1* status	*IDH1* mutant	Adjuvant therapy	Radiation	1	(33.3%)	2	(66.7%)	0.476^b^
			Chemoradiotherapy	5	(17.2%)	24	(82.8%)	
	*IDH1* wildtype		Radiation	11	(64.7%)	6	(35.3%)	0.197^a^
			Chemoradiotherapy	15	(45.5%)	18	(54.5%)	
*MGMT* status	Methylated	Adjuvant therapy	Radiation	1	(50.0%)	1	(50.0%)	0.999^b^
			Chemoradiotherapy	9	(34.6%)	17	(65.4%)	
	Unmethylated		Radiation	11	(61.1%)	7	(38.9%)	0.031^a^
			Chemoradiotherapy	11	(30.6%)	25	(69.4%)	
*IDH1* status	*IDH1* mutant	Chemotherapy	TMZ	5	(23.8%)	16	(76.2%)	0.283^b^
			TMZ plus others	0	(0.0%)	8	(100.0%)	
	*IDH1* wildtype		TMZ	15	(60.0%)	10	(40.0%)	0.118^b^
			TMZ plus others	2	(22.2%)	7	(77.8%)	
*MGMT* status	Methylated	Chemotherapy	TMZ	8	(42.1%)	11	(57.9%)	0.666^b^
			TMZ plus others	2	(25.0%)	6	(75.0%)	
	Unmethylated		TMZ	12	(44.4%)	15	(55.6%)	0.016^b^
			TMZ plus others	0	(0%)	9	(100.0%)	

*Data are shown as n (%).

^a^ *p*-Values of Chi-Square test.

^b^ *p*-Values of Fisher’s Exact test.

### Relationship of IDH1 Mutation or MGMT Promoter Methylation and Adjuvant Therapies With Recurrence-Free Interval in Glioblastoma Patients

There was no significant difference in tumor recurrence among patients treated with any adjuvant therapy protocol regardless of *IDH1* status (Table 3). However, a difference was clearly seen among glioblastoma patients with unmethylated *MGMT* promoter who were treated with radiation vs. those who received chemoradiotherapy (*p*-value = 0.031) (Figure 2B). Among patients with *MGMT* promoter methylation, there was no significant difference in tumor recurrence interval regardless of the type adjuvant therapy.

### Relationship of IDH1 Mutation or MGMT Promoter Methylation and the Type of Chemotherapy With Recurrence-Free Interval in Glioblastoma Patients

**FIGURE 3:**
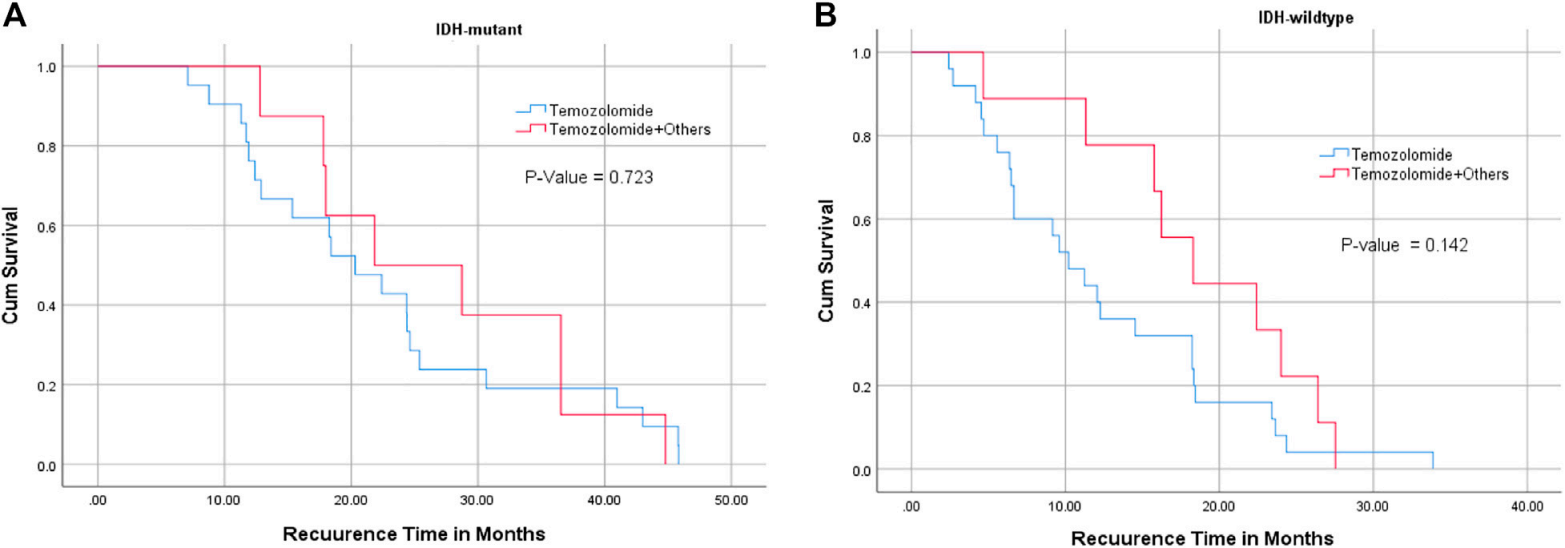
Relationship between *IDH1* mutation status (**(A)** : IDH mutant, **(B)**: IDH wildtype), chemotherapy, and recurrence-free interval. TMZ alone or combined with chemotherapies did not significantly affect tumor recurrence interval in *IDH1* mutant and wildtype glioblastoma patients.

There was no significant relationship between the type of chemotherapy and recurrence-free interval in *IDH1* mutant or wildtype glioblastoma cases (Figure 3) (Table 3). However, patients with *MGMT* promoter methylation showed significant differences in the recurrence-free interval. In patients with unmethylated *MGMT* promoter, those treated with TMZ and other chemotherapeutic agents showed late recurrence compared with those who were treated with TMZ alone (*p*-value = 0.014) (Tables 3–5).

**TABLE 4:** The association between *IDH1* mutation and *MGMT* methylation status with recurrence-free interval within 3 years among glioblastoma patients treated with radiotherapy or chemoradiotherapy.

					95% CI	
	Period in months	Number risk at end of interval	Number of cases recurred	Recurrence free %	Lower	Upper
*IDH1* mutant						
Radiation	12	2	1	66.7%	30.0%	100.0%
Radiation	24	2	0	66.7%	30.0%	100.0%
Chemoradiotherapy	12	24	5	82.8%	70.1%	97.7%
Chemoradiotherapy	24	13	11	44.8%	29.9%	67.1%
*IDH1* wildtype						
Radiation	12	6	11	35.3%	18.5%	67.2%
Radiation	24	3	3	17.6%	6.3%	49.3%
Chemoradiotherapy	12	19	14	57.6%	43.0%	77.2%
Chemoradiotherapy	24	6	13	15.2%	6.8%	34.0%
*MGMT* methylation						
Radiation	12	1	0	100.0%	100.0%	100.0%
Radiation	24	1	0	100.0%	100.0%	100.0%
Chemoradiotherapy	12	9	1	90.0%	73.2%	100.0%
Chemoradiotherapy	24	7	2	70.0%	46.7%	100.0%
Chemoradiotherapy	36	4	3	40.0%	18.7%	85.5%
Unmethylated *MGMT*						
Radiation	12	1	1	50.0%	12.5%	100.0%
Radiation	24	1	0	50.0%	12.5%	100.0%
Chemoradiotherapy	12	15	4	78.9%	62.6%	99.6%
Chemoradiotherapy	24	6	9	31.6%	16.3%	61.2%
Chemoradiotherapy	36	3	3	15.8%	5.6%	44.6%

Levels					95% CI	
	Time	Number risk at end of interval	Number of cases recurred	Recurrence free %	Lower	Upper
Unmethylated *MGMT IDH1* mutant						
Temozolomide	12	9	4	69.2%	48.2%	99.5%
Temozolomide	24	4	5	30.8%	13.6%	69.5%
Temozolomide	36	2	2	15.4%	4.3%	55.0%
Temozolomide + Others	12	6	0	100.0%	100.0%	100.0%
Temozolomide + Others	24	2	4	33.3%	10.8%	100.0%
Temozolomide + Others	36	1	1	16.7%	2.8%	99.7%
Unmethylated *MGMT IDH1* wildtype						
Temozolomide	12	7	7	50.0%	29.6%	84.4%
Temozolomide	24	2	5	14.3%	4.0%	51.5%
Temozolomide + Others	12	3	0	100.0%	100.0%	100.0%
Temozolomide + Others	24	1	3	0.0%	Na	Na

CI: confidence interval; Na, not applicable.

**TABLE 5:** The association between IDH1-mutation, MGMT methylation and recurrence-free interval (days) in patients receiving TMZ with additional adjuvant chemotherapies.

IDH1 status	MGMT status	Chemotherapies types	RI
IDH-mutant	MGMT hypermethylation	TMZ + bevacizumab	1344
IDH-wildtype	MGMT hypermethylation	TMZ + bevacizumab	140
IDH-mutant	MGMT hypermethylation	TMZ + irenotecan + bevacizumab	1096
IDH-wildtype	Unmethylated	TMZ + bevacizumab	720
IDH-wildtype	MGMT hypermethylation	TMZ + bevacizumab	340
IDH-wildtype	MGMT hypermethylation	TMZ + bevacizumab	672
IDH-wildtype	MGMT hypermethylation	TMZ + irenotecan + bevacizumab	792
IDH-mutant	Unmethylated	TMZ + lomustine + bevacizumab	862
IDH-wildtype	Unmethylated	TMZ + bevacizumab	487
IDH-mutant	Unmethylated	TMZ + bevacizumab	540
IDH-wildtype	MGMT hypermethylation	TMZ + lomustine + bevacizumab	473
IDH-wildtype	MGMT hypermethylation	TMZ + bevacizumab	826
IDH-mutant	Unmethylated	TMZ + bevacizumab	1096
IDH-mutant	Unmethylated	TMZ + lomustine	655
IDH-wildtype	Unmethylated	TMZ + bevacizumab + etoposide	1004
IDH-wildtype	Unmethylated	TMZ + bevacizumab	549
IDH-mutant	Unmethylated	TMZ + bevacizumab	534

*RI: Recurrence interval.

### Recurrence-Free Intervals Between Different Groups

*IDH1*-mutant cases treated with only radiotherapy were 66.7% recurrence free at 12 months of treatment while *IDH1*-mutant cases who received chemoradiotherapy were 82% recurrence free at 12 months (Table 4). This clarified that chemoradiotherapy had a better beneficial outcome. In *IDH1*-wildtype glioblastoma cases treated with chemoradiotherapy showed 57.6% recurrence free in the 12^th^ month, which is relatively higher than what was observed in *IDH1*-wildtype cases that received only radiation (35.3%), however; slightly opposite behavior was observed at 2 years. Another example, cases with *MGMT* promoter methylation treated with chemoradiotherapy showed recurrence free of 90%, 70% and followed by 40% at 1, 2, and 3 years, respectively. On the other hand, cases with unmethylated *MGMT* treated with chemoradiotherapy had recurrence free of (78.9, 31.6, and 15.8%) at 1, 2, and 3 years, respectively. This interpretation was quite clear also for the type of chemotherapies used in the treatment of glioblastoma (See Table 4).

## Discussion

Glioblastoma is the most aggressive primary malignant brain tumor in adults [1]. Both primary and secondary glioblastomas are pathologically indistinguishable, but they vary at the molecular level [2]. The disease remains fatal although the palliative treatment modalities given to the patients [2, 3]. These treatments are currently dependent on several molecular markers including *IDH1* mutation and *MGMT*-gene promoter methylation. Despite the controversy on the impact of these biomarkers, they are still considered prognostic factors in patients with glioblastoma. While wildtype *IDH1* is present in 90% of primary glioblastomas [1], mutant *IDH1* is common in secondary glioblastomas and associated with increased patient survival [4, 6]. Epigenetic-mediated silencing of the *MGMT* gene in glioblastoma by promoter methylation has also been shown to correlate with better OS. Use of *IDH1* combined with *MGMT* promoter as a stratification factor seems appropriate in clinical trials for the treatment of patients with secondary glioblastoma [6].

In the present analysis, we evaluated the impact of *MGMT* promoter methylation as well as *IDH1* mutation status on recurrence-free interval (the period from beginning of adjuvant therapy after surgical resection to the possible first date of recurrence) in patients with glioblastoma. We used recurrence-free interval (RI) rather than OS as RI is more accurate and also doesn’t provide false predictability in the outcome of glioblastoma patients.

Because our results found that there was no significant relationship between *IDH1* mutation and *MGMT* promoter methylation, both factors are considered prognostically independent (Table 2). This relationship can be considered significant if the age factor was included in the prognostic analysis. For example, we found that more glioblastoma patients younger than 50 years old with *IDH1* mutant tumors had tumor recurrence after one year of treatment compared with those with *IDH1* wildtype (Figure 2A; Table 3).

The impact of *MGMT* promoter methylation status in patients with glioblastoma also showed controversial results. Hegi et al. showed that glioblastomas with methylated *MGMT* promoters were more sensitive to chemotherapeutic agents, including TMZ, resulting in an OS benefit for these patients [9]. Dunn et al showed that a greater extent of methylation was associated with significantly longer OS [10]. Other studies in anaplastic gliomas have shown that *MGMT* promoter methylation status did not only influence patient outcome after chemotherapy but also impacted outcome after radiotherapy and may therefore be prognostic rather than predictive [11]. In contrast, other studies revealed that *MGMT* promoter methylation was not associated with better OS and progression-free survival; both endpoints were comparable in patients with expressed *MGMT* and those with *MGMT* silencing. The studies by Costa et al. and Park et al. on glioblastoma patients treated with TMZ-based chemoradiation revealed that *MGMT* promoter methylation was not associated with improved outcome [12, 13]. Our study found that *MGMT* promoter methylation was not associated with a significant change in patient outcome. However, glioblastomas with unmethylated *MGMT* promoter, who received chemoradiotherapy mainly TMZ therapy, had a late tumor recurrence (Figure 2B; Table 3). Hence, *MGMT* promoter methylation in glioblastoma cannot be considered as a significant factor for long survival.

The relationships between *IDH1* mutation and *MGMT* promoter methylation with the type of chemotherapies were also assessed independently with tumor recurrence interval. Our results found that glioblastoma cases, regardless of *IDH1* status, that were treated with TMZ or TMZ and additional chemotherapeutic agents did not show any difference in tumor recurrence interval (Figure 3). This was completely different among cases in which *MGMT* methylation was the main independent factor. Although methylated *MGMT* promoter with *IDH1* mutant glioblastoma cases showed clinically significant results, the cases with unmethylated *MGMT* promoter and *IDH1* mutation treated with combined chemotherapies had significantly late tumor recurrence. These cases were mostly *IDH1* mutant (Table 4). This means that glioblastoma with wildtype *IDH1* and unmethylated *MGMT* promoter should be treated aggressively with combined chemotherapies (TMZ plus other chemotherapies) to improve overall survival. Two other studies also found conflicted findings. Combs et al. analyzed a group of 160 patients with glioblastoma treated with radiotherapy and TMZ for the impact of *MGMT* promoter methylation and *IDH1* mutation. Unexpectedly, OS was no longer among the group of methylated *MGMT* promoter compared with patients without *MGMT* promoter methylation [17]. In contrast, Millward et al. demonstrated that the combination of methylated *MGMT* and *IDH1* mutation was associated with considerably longer OS and PFS in a series of chemoradiotherapy-treated glioblastoma tumors [14]. Alassiri et al. also found a positive impact of both *MGMT* methylation and *IDH1* mutation on the overall survival of Saudi patients with glioblastoma [18] but the study did not relate the findings with other treatment modalities. Another recent study by Pandith et al investigated the relationship between IDH1/IDH2 mutations and MGMT methylation in patients receiving chemotherapies in different types of malignant gliomas (astrocytomas and oligodendrogliomas). Compared to our study, the only treatment modality used in their study was TMZ chemotherapy while IDH1/2 sequencing was the primary method to detect mutation. Using IDH1/IDH2 sequencing from fresh tissue nowadays are somewhat difficult either because of limited facilities or because the high cost of sequencing [19]. Our results concluded that *IDH1* mutation and *MGMT gene* promoter methylation are considered independent prognostic factors in glioblastoma. Although *IDH1* mutant glioblastomas showed late tumor recurrence in patients younger than 50 years of age, the type of treatment modalities may not show additional beneficial outcome. Patients with unmethylated *MGMT* gene promoter with *IDH1* mutation, treated with chemoradiotherapy including TMZ had a late tumor recurrence while glioblastomas with wildtype *IDH1* and unmethylated *MGMT* gene promoter should be treated aggressively with radiotherapy and combined chemotherapies to delay tumor recurrence.

One limitation must be acknowledged in our study, that the total number of cases analyzed for *MGMT* promoter methylation and *IDH1* mutation are low. Despite this limitation, our report is the first study in Saudi Arabia that correlates these molecular biomarkers with recurrence-free interval in totally resected glioblastomas, reflecting the impact of adjuvant therapies as well as the specific type of chemotherapies on patient outcome.

## Conclusion

This is the first paper to describe the role of IDH1 mutation and MGMT promoter methylation status in terms of overall survival and recurrence-free survival by patients group treated with different therapeutical modalities in Saudi Arabia. The impact of IDH mutations and MGMT promoter methylation on OS and PFS is extensively described in the literature. Our results highlighted the impact of these molecular biomarkers on different treatment modalities including radiotherapy and chemotherapies on glioblastoma outcome. Additional molecular studies and raising the sample size could increase the value of the investigation.

## Data Availability

The data that support the findings of this study are available from the corresponding author upon request.
